# Gingival hyperplasia as first sign of recurrence of granulomatosis with polyangiitis (Wegener’s granulomatosis): case report and review of the literature

**DOI:** 10.1186/s12903-016-0262-4

**Published:** 2016-08-02

**Authors:** Marcel Hanisch, Leopold F. Fröhlich, Johannes Kleinheinz

**Affiliations:** Department of Cranio-Maxillofacial Surgery, University Hospital Münster, Albert-Schweitzer-Campus 1, Gebäude W 30, D-48149 Münster, Germany

**Keywords:** Granulomatosis with polyangitis, Orofacial manifestation, Recurrence, Rituximab, Strawberry gingivitis, Wegener’s granulomatosis

## Abstract

**Background:**

Granulomatosis with polyangiitis (GPA), formerly referred to as Wegener’s granulomatosis, is a rare systemic disease of unknown etiology which can affect all areas of the body, including the oral cavity. The typical oral manifestations occur as nonspecific erosive/ulcerative lesions of the oral cavity or appear with hyperplastic gingivitis, a so called “strawberry gingivitis”.

**Case presentation:**

We report here about an extremely rare case with hyperplastic gingivitis as the first sign of recurrence of GPA in the absence of oral manifestations in the primary disease. A 72 year-old female was referred to our Department of Cranio-Maxillofacial Surgery with hyperplastic gingivitis. The patient was diagnosed with GPA already eight years before. We referred the patient to our Clinic of Internal Medicine where she was successfully treated with rituximab. At the follow-up visit, the patient showed complete remission of the hyperplastic gingiva.

**Conclusion:**

The often overlooked oral manifestation may be interpreted as the first evidence of resurgent GPA in general and therefore could be pathognostic for the disease. This case affirms the need of health professionals to be acquainted with orofacial manifestations of rare diseases such as GPA. As a consequence, dentists will be able to assist in diagnosing GPA more easily leading to a better prognosis for patients suffering from this disease.

## Background

GPA, formerly referred to as Wegener’s granulomatosis, is a rare, systemic disease of unknown etiology characterized by necrotizing granulomatous inflammation of the upper and lower respiratory tract, glomerulonephritis and vasculitis [1]. Moreover, nasal deformations with saddle nose manifestation, sinusitis and ulceration of the palate or nasal mucosa are not extraordinary in GPA. General GPA can affect all areas of the body, including the oral cavity [2, 3]. In European Union countries, any disease affecting less than one person in 2.000 is considered “rare” [4]. GPA has a prevalence of 1–9/100.000 and therefore is listed as a rare disease [5]. About 5.000 rare diseases are of genetic origin and approximately 15 % of these present manifestations at the orofacial region [6]. Oral symptoms in GPA have been reported in 10–62 % [7]. The typical oral manifestations occur as nonspecific erosive/ulcerative lesions of oral cavity or appear with hyperplastic gingivitis, a so-called “strawberry gingivitis”. GPA can affect patients at any age, whereas diagnosis is mostly done when patients range between 40 and 55 years of age. The disease is more common in Caucasians and no gender-specific differences were identified [8]. The pathogenetic cause of GPA is mediated by a T-cell reaction leading to the production and release of pro-inflammatory cytokines like TNF-α and IFN-γ which induce the expression of surface antigens on activated neutrophil granulocytes. One of these antigens is proteinase 3 which is the target of antineutrophil cytoplasmatic antibodies (c-ANCA). This interaction leads to degranulation of neutrophil granulocytes which release proteases and effector molecules being responsible for the tissue damage [9]. Generally, the clinical manifestation of the disease varies from patient to patient. Rapid progression in combination with multiorgan failure will lead to death if untreated [10]. Two types of GPA are known: diffuse forms, manifesting primarily by renal and pulmonary contribution and localized forms, limited to the upper respiratory tract. Localized forms are in contrast to diffuse forms more recurrent while diffuse forms are initially more fatal but less recurrent [11, 12]. A transition from limited to localized GPA and the other way around is possible [12]. Since the disease develops over an extended period of time, it usually takes 4.7–15 months from the beginning of the symptoms to the diagnosis [8]. The American College of Rheumatology proposes that for the diagnosis of “GPA” two or more of the following criteria have to be fulfilled: (1) ulcerative lesions in the oral mucosa or nasal bleeding or swelling, (2) nodules, infiltrates or cavities on chest radiograph, (3) abnormal urinary sediment, or (4) granulomatous inflammation on biopsy [13]. Here, we report about an extremely rare case with gingival hyperplasia as the first sign of recurrence of GPA in the absence of oral manifestations in the primary disease.

## Case presentation

A 72 year-old female was transferred to our Department of Cranio-Maxillofacial Surgery at the University Hospital of Münster with a hyperplastic gingivitis and intraoral pain in October 2015.

### Patient history

The patient was diagnosed with GPA already in 2007. Since that time she has developed glomerulonephritis, nephrosclerosis, pulmonary emphysema, Raynaud’s phenomenon, sinusitis maxilliaris and had surgery on an orbital pseudotumor in 2014. Since the first diagnosis of GPA in 2007 the patient was treated with prednisolone and cyclosporine. She also suffered from a steroid-associated osteoporosis which has been treated intravenously with denosumab, a human monoclonal antibody directed towards osteoclast-mediated bone resorption by binding to osteoblast-produced RANKL. Mild hypertonia and presbyacusia of both ears were also diagnosed. Oral manifestations like “strawberry gingivitis” did not occur since the first diagnosis of GPA in 2007, but a basal cell carcinoma developed in 2015. The patient was first diagnosed with hyperplastic gingivitis by her dentist in April 2015 and was treated subsequently by non-surgical periodontal therapy without antibiotics. However, since the therapy lacked any beneficial effect the patient was sent to a periodontist in September 2015. The periodontist did not conduct any treatment. He recommended gingivectomy of the hyperplastic gingiva and due to the additional intravenous denosumab therapy, he transferred the patient to our Department for further treatment.

### Oral examination

**Fig. 1:**
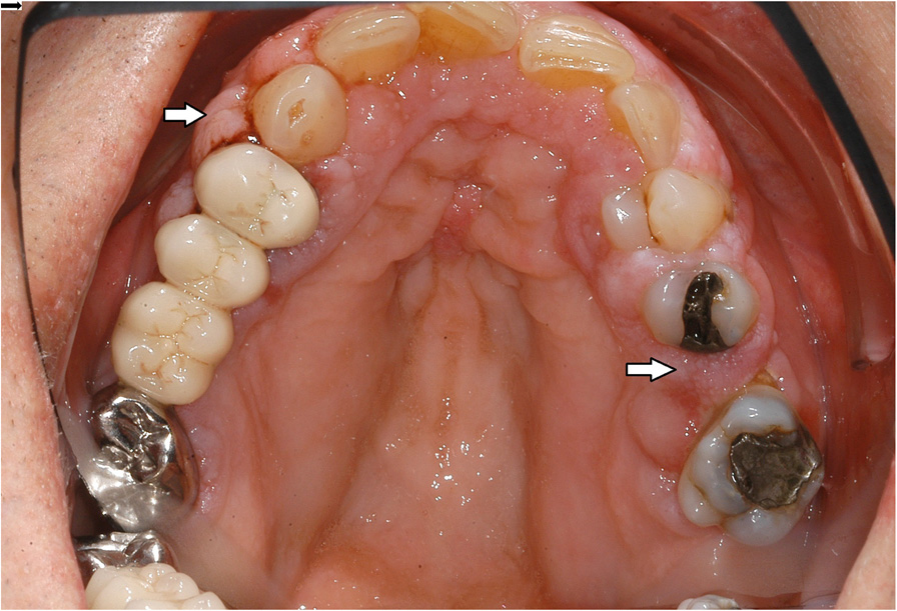
Photographical Image of oral lesion at initial presentation

**Fig. 2:**
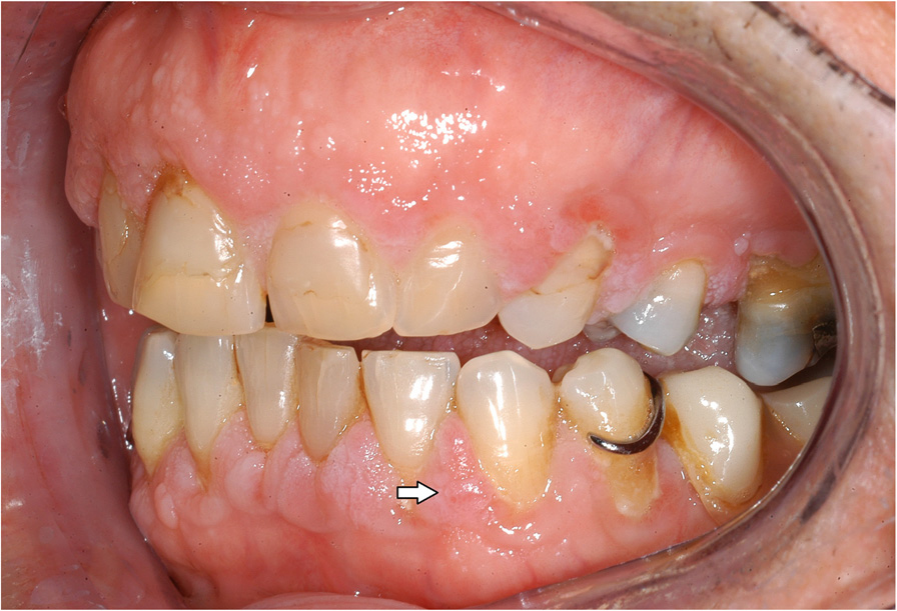
Photographical image of the oral lesion at initial presentation

**Fig. 3:**
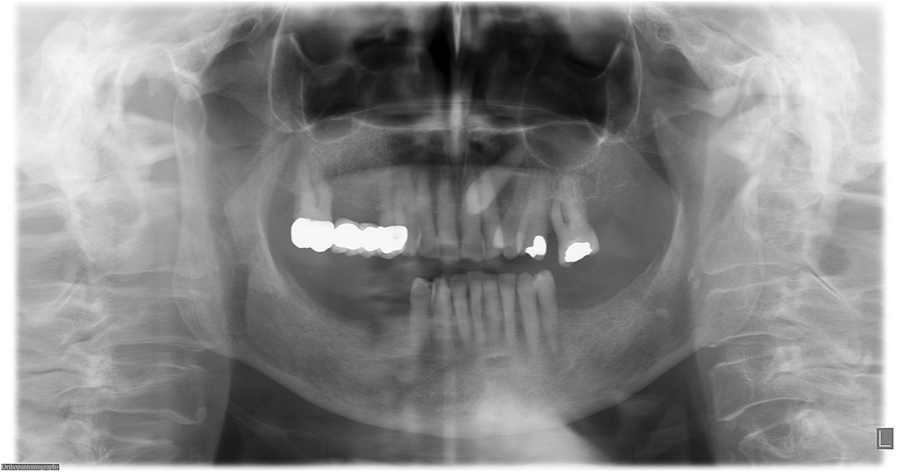
Panoramic radiograph with impacted canine, furcation involvment of the upper molars and horizontal bone loss according to the age

In the intraoral examination, alterations of the gingival volume were observed. Plaque control was poor since handling with the toothbrush was too difficult for the patient. Additionally, we noted the absence of petechia on the buccal and oral gingiva in the upper as well as in the lower jaw and bleeding in the buccal area of 13–15. (Figs. 1 and 2). She described intraoral pain and was handicapped during food intake. In the orthopantomogram the patient showed an impacted canine, furcation involvement of the first upper molars, and horizontal bone loss according to the age (Fig. 3). Thus, the gingival volume appeared uncommon.

### Present medication

At the time of the patient’s visit at our Department the medication for the therapy of GPA consisted of a combination of prednisolone 10 mg/day and cyclopsporine 150 mg/day prescribed by her family doctor. The patient’s full medication is presented in Table 1.

**Table 1:** Patient’s full medication

Medication	Dosage per day
Amlodipine	10 mg
Flecainide acetat	50 mg
Xipamide	5 mg
Prednisolone	10 mg
Ciclosporine	150 mg
Omeprazole	40 mg
L-Thyroxine	125 *μg*
Metamizole	500 mg
Vitamin D3	1200 mg

### Therapy

**Fig. 4:**
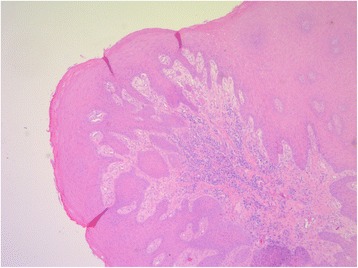
Histology of chronic, florid inflammation with stratified epithelium, parakeratosis, increased capillarization and intraepithelial neutrophilic granulocyte infiltration stained with hematoxylin and eosin (PAS, magnification: 100-fold)

**Fig. 5:**
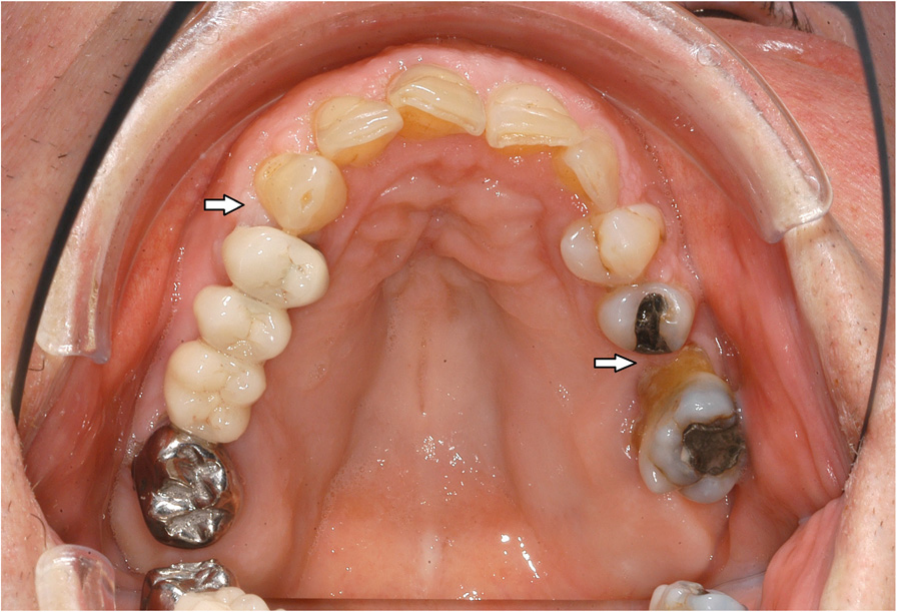
Resolution of the oral lesion in Fig. 1 following therapy with rituximab

**Fig. 6:**
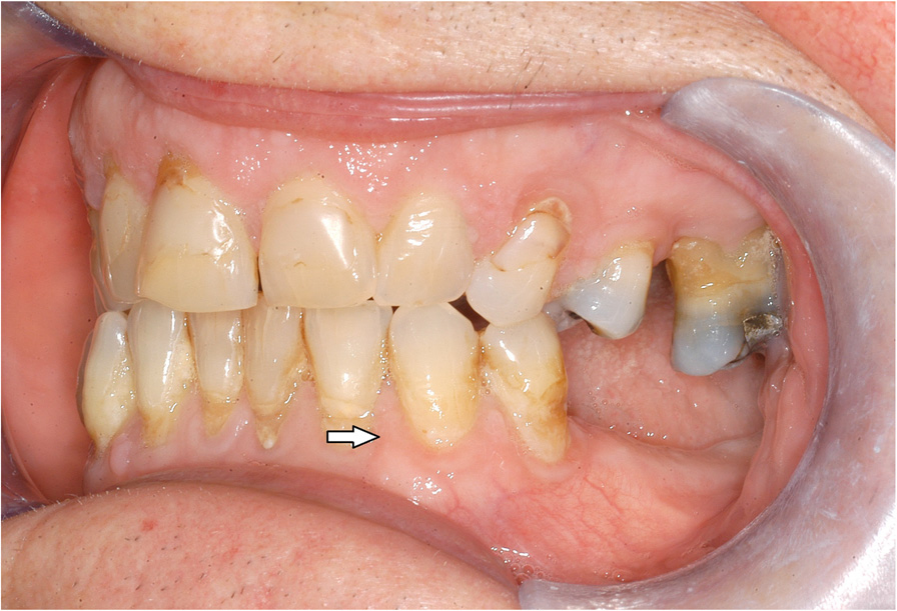
Resolution of the oral lesion in Fig. 2 following therapy with rituximab

As periodontal therapy has previously failed and cyclosporine has been given since 2007 without any intraoral side-effects like gingival hyperplasia, we decided in suspicion of the recurrence of GPA with gingival hyperplasia to take a biopsy from the palatinal premolar region under local anesthesia. The biopsy was then sent for routine histopathological analysis which revealed inflammation with parakeratosis and neutrophil-granulocytic infiltration (Fig. 4). These findings were conveyed to her family doctor who decided to refer the patient to the Clinic of Internal Medicine at the University Hospital of Münster. Since the staining for c-ANCA tested positive, the recurrence of GPA was suspected. Therefore, they decided to treat the patient with rituximab via infusion. In addition to prednisolone 10 mg/day, the patient was given 375 mg/m^2^ rituximab weekly for a total of 4 doses. After one week of therapy, she was re-evaluated at our Department. At the follow-up visit, the patient showed complete remission of the hyperplastic gingiva (Figs. 5 and 6), the absence of pain, and the patient was able to eat without problems. Periodontal probing depths of 5 mm or more only appeared at the molars. Table 2 shows the diagnostic and treatment process of the patient in chronological order.

**Table 2:** The patient’s diagnostic and treatment process in chronological order

2007	2007–2015	April-October 2015	October 2015-February 2016	December 2015-January 2016
First diagnosis of GPA diagnosed by glomerulon ephritis	Permanent medication with prednisolone and cyclosporine. Development of a steroid-associated osteoporosis treated intravenously with denosumad. Over the time developing pulmonary emphysema, Raynaud’s phenomenon, sinusitis maxilliaris and orbital pseudotumor.	Development of gingival hyperlpasia. In the following periodontal theraphy without benefit by her dentist. Thus reffered to periodontist. Recommendation for gingivectomy, transfer to Department of Cranio-Maxillofacial Surgery, University Hospital Münster. Biospsy from hyperplastic gingivitis taken	Ambulant follow-up in the Department of Cranio-Maxillofacial Surgery, University Hospital Münster.	Suspiction of recurrence of GPA by increasing (c)-ANCA and clinical signs of recurrence. Stationary therapie at Internal Medicine Clinic, University Hospital Münster. In the following 375 mg/m^2^ rituximab weekly for a total of 4 doses, and prednisolone 10 mg/day.

## Discussion

Cases of GPA with oral symptoms were described in several reports [2, 3, 7, 14–18]. However, a systematic search in PubMed generated by “granulomatosis with polyangiitis, Wegener’s granulomatosis, gingivitis, recurrent, recurrence” indicated only one report describing the recurrence of GPA without any oral symptoms in the initial presentation of the disease reported by Staines et al. [14]. Comparable to our case, gingival hyperplasia was evident only on recurrence in this single reported case. The standard therapy of initial GPA consisted of glucocorticoids or cyclophosphamide, or a combination of both [19]. Although standard therapy does improve the survival rate of the patients, there are also therapy-related malignancies like squamous cell carcinoma, Kaposi sarcoma or basal cell carcinoma which was also applicable to our patient [20]. In contrast to the standard therapy of initial GPA, there are reports of successful treatment of refractory head and neck GPA with rituximab [14]. Rituximab is a chimeric, monoclonal antibody targeting the CD20-antigen expressed by B-cells. Successful treatment with rituximab leading to full remission is reported in 62 % of all treated patients by Martinez et al. [21]. About 15 % of all rare diseases show orofacial manifestations [6, 22]. Typical oral manifestations in GPA are unspecific erosive lesions in the oral mucosa or hyperplastic gingivitis as reported here. Differential diagnosis for the gingival enlargement could be caused by medications [2] (such as phenytoin, cyclosporine and some oral contraceptives), other granulomatous diseases like sarcoidosis and Crohn’s disease or blood dyscrasia like leukemia [7]. Since oral lesions can be localized a long time before multiorgan involvement actually occurs [15], the often overlooked oral manifestation may be interpreted as the first sign of GPA and could be pathognostic for the disease. Histopathological pattern of GPA include granulomatous inflammation, vasculitis, necrosis and multinucleated giant cells. Often, however, histopathological findings are less specific and cannot be considered as clinical diagnostics [16]. Furthermore, it is known that rare diseases with oral components benefit more from an early diagnosis than rare diseases without oral components [23]. Misdiagnosed oral manifestations which are often overlooked by physicans and by dentists may be interpreted as the first evidence of resurgent GPA in general and therefore could be pathognostic for the disease. A dental specialist has a high chance of identifying GPA based on the characteristics of the oral findings. Moreover, the dentist might be the first one being consulted by the patient with oral manifestations such as gingival hyperplasia. Gingival hyperplasia that is not associated with remission after periodontal therapy or with drug delivery should be investigated then with regard to internal diseases like GPA or leukemia. This example affirms why health professionals should be acquainted with orofacial manifestations of rare diseases such as GPA. As a consequence faster referrals to other medical specialists like rheumatologist, nephrologist, or pulmonologist will be possible and enable them to start with the treatment early on, on the one hand [17]. On the other hand, dentists may receive referrals from physicans for evaluating oral symptoms.

## Conclusions

Overall, this case demonstrates an important role for the dentist in the early diagnosis of GPA. Instead of gingivectomy with conceivably fatal consequences, treatment of the underlying disease based on oral symptoms was performed. In conclusion, dentists should be acquainted with orofacial manifestations of rare diseases such as GPA, because they could be the first in detecting early symptoms of a rare disease. Thus, the dentist will be able to assist in the early diagnosis of this fatal rare disease more easily which is the most important factor in the management and prognosis for patients suffering from GPA or other rare diseases [18].

## Abbreviations

c-ANCA, antineutrophil cytoplasmatic antibodies; GPA, granulomatosis with polyangiitis
